# Reducing Pre- and Post-Treatments in Cryopreservation Protocol and Testing Storage at −80 °C for Norway Spruce Embryogenic Cultures

**DOI:** 10.3390/ijms232415516

**Published:** 2022-12-08

**Authors:** Saila A. Varis, Susanna Virta, Itziar A. Montalbán, Tuija Aronen

**Affiliations:** 1 Natural Resources Institute Finland (Luke), Vipusenkuja 5, FI-57200 Savonlinna, Finland; 2 Department of Forestry Science, NEIKER, 01080 Arkaute, Spain

**Keywords:** abscisic acid, long-term storage, polyethylene glycol, *Picea abies*, pre-treatment, somatic embryogenesis, sucrose concentration, ultra-low temperature freezing

## Abstract

Somatic embryogenesis (SE) is considered the most effective method for vegetative propagation of Norway spruce (*Picea abies* L. Karst). For mass propagation, a storage method that is able to handle large quantities of embryogenic tissues (ETs) reliably and at a low cost is required. The aim of the present study was to compare freezing at −80 °C in a freezer to cryopreservation using liquid nitrogen (LN) as a method for storing Norway spruce ETs. The possibility of simplifying both the pre-treatment and thawing processes in cryopreservation was also studied. The addition of abscisic acid (ABA) to the pre-treatment media and using polyethylene glycol PEG4000 instead of PEG6000 in a cryoprotectant solution were tested. Both the pre-and post-treatments on semi-solid media could be simplified by reducing the number of media, without any loss of genotype or embryo production capacity of ETs. On the contrary, the storage of ETs in a freezer at −80 °C instead of using LN was not possible, and the addition of ABA to the pre-treatment media did not provide benefits but increased costs. The lower regeneration rate after using PEG4000 instead of PEG6000 in a cryoprotectant solution in cryovials was unexpected and unwanted. The simplified pre-and post-treatment protocol will remarkably reduce the workload and costs in the mass-cryopreservation of future forest regeneration materials and in thawing the samples for mass propagations, respectively.

## 1. Introduction

The demand for forest biomass has increased due to the growing need for substitutes for fossil fuels and the conservation of more natural forests with high biodiversity [1,2]. To increase forest growth and thus increase the possibility of their sustainable use, the best possible regeneration material, i.e., superior tree genotypes, should be used [3]. To ensure the availability of good-quality forest regeneration material, effective vegetative propagation methods such as somatic embryogenesis (SE) have been introduced [4]. SE has become the method of choice for the vegetative propagation of conifers due to its high multiplication rate and the maintenance of juvenility via cryopreservation in liquid nitrogen (LN), which allows the long-term field testing of materials [4,5]. When the aim is commercial production, the process needs scaling up, and this includes increasing the size of the cryo-bank [6]. In Finland, a research program has been launched to produce material for future forestry by applying SE methods, including the cryopreservation of embryogenic tissue (ET) from thousands of Norway spruce (*Picea abies* (L.) Karst.) genotypes [7,8,9]. Many different genotypes are needed to maintain genetic diversity in reforestation, as is also suggested for the vegetative propagation approach utilizing SE in Finland [9,10]. However, the production of SE plants is more expensive than seedling production due to the amount of skill-intensive manual labor and laboratory conditions required [11]. For the mass propagation program, the cryopreservation method applied should therefore be able to handle large numbers of samples at a low cost but with high reliably [6].

Most plant samples, such as ET and embryos, contain a large amount of intra- and extracellular water. This means they are vulnerable to injuries such as crystallization during fast freezing [12]. Cryopreservation protocols based on cryoprotectants with both classical slow cooling [13,14,15,16,17] and vitrification techniques [18,19] have been developed for embryogenic cultures of different spruce species. More recently, cryopreservation protocols based on the drying of ET [20,21], immature [22], or mature somatic embryos [23,24] of various spruce species and their direct immersion in liquid nitrogen (LN) without the use of cryoprotectants have also been described. Various treatment combinations have been used to dry the ET of conifers prior to cryopreservation. In an early version, Gupta et al. [25] used increasing glucose concentrations in suspension cultures also containing polyethylene glycol (PEG) and dimethylsulfoxide (Me_2_SO) as cryoprotectants. Klimaszewska et al. [16] and Find et al. [14] used sorbitol instead of glucose. Hazubska-Przybyl et al. [21] introduced the dehydration of Norway spruce, increasing the sucrose concentration (0.25 M to 1.0 M) in semi-solid media with a low concentration of abscisic acid (ABA) and desiccation using silica gel. The ET was then frozen without cryoprotectants directly to LN [21]. Varis et al. [8] gained 87% recovery with Norway spruce ET after dehydrating them on semi-solid medium with sucrose concentrations of 0.1 M and 0.2 M, applying a mixture of PEG 6000, glucose, and Me_2_SO, and slow-cooling at a rate of 0.17 °C/min.

ABA is a key endogenous messenger and the central regulator of abiotic stress resistance in plants, and it coordinates an array of functions [26]. Exogenous ABA has been used in cryopreservation to increase tolerance to oxidative stress during freezing and improve plant regeneration [26]. ABA has also been successfully used in the cryopreservation of coniferous species [20,21,22]. PEG is an osmotic synthetic polymer which can be used as a dehydrative agent in SE in maturation or in preservative liquid in cryovial.

Cryopreservation is rather expensive due to the high price of LN and the special containers it requires. Storing at −80 °C could reduce the expense, although the price of electricity is increasing. In this sense, Montalbán and Moncaleán [27] succeeded in storing the ET of radiata pine (*Pinus radiata*) using an ultra-low temperature freezer. The ET was dehydrated in a suspension culture with a sucrose content of 0.53 M and supplemented with Me_2_SO, after which samples were immersed in LN for 5 min. After one year’s storage, the recovery percentage and plant conversion ability were both above 75%.

Thawing of the samples from cryopreservation has gained less attention than pre-treatment. Most protocols involve short heating at a low temperature in a water bath and removing the content of the cryovials into a filter-placed medium used in ET proliferation [14,16] or supplemented by a decreasing concentration of sucrose [8,21]. The proliferation medium is usually refreshed at least once, meaning the workload is similar in both methods.

This study’s aim was to assess whether it was possible to store embryogenic cultures of Norway spruce originating from the elite trees of the Finnish tree breeding program at −80 °C in the long term without a loss of regeneration capacity. The pre-treatment and slow-cooling technique that has this far shown the best recovery rates in the species was chosen as the cryopreservation method, and the same technique was tested with freezing at −80 °C. The pre-treatment method in a liquid medium was tested with slow-cooling in a programmable freezer or Mr. Frosty^®^ (i.e., Nalgene’s container designed to hold cryovials during slow cooling). Another aim was to study if it was possible to simplify the previously used cryopreservation procedure using only one pre-treatment and post-thawing medium with elevated sucrose content instead of two. ABA was also added to the pre-treatment media to evaluate the effect on the regeneration of cryopreservation materials and embryo maturation.

## 2. Results

### 2.1. Experiment I to Test ABA in the Pre-Treatment Media

When ABA was added to the pre-treatment media, the regeneration percentage of the samples was 56%; in control samples it was 78%. At least two samples from six out of nine SE lines regenerated when pre-treated with ABA, compared to eight lines from the control media (Table 1). Moreover, only five lines produced enough ET for maturations when they were pre-treated with ABA (56.66 ± 15.94 E/g FW), but every line produced at least some embryos. Of the controls, only three SE lines produced embryos. Although there were slightly more embryos, 61.72 ± 19.14 E/g FW, than in the ABA pre-treated lines, the difference was not significant. Before cryopreservation, the embryo productivity of the same five lines was 87.86 ± 32.38 E/g FW.

### 2.2. Experiment II to Test Storage at −80 °C with Several Pre-Treatment and Slow-Cooling Methods

After three months’ storage, the viability of samples which were cryopreserved using pre-treatment on solid media and freezing in a Planer device was 90% and 23% when the same pre-treatment and slow-cooling methods were used, but the samples were stored at −80 °C. The recovery percentages from the same treatments were 85 and 18 after ten months’ storage. Because of the clear regeneration results, the embryo productivity was not tested. All the samples pre-treated in a suspension and stored at −80 °C showed no growth after thawing and two months’ observation.

### 2.3. Experiment III to Test Reduction of Post-Thawing Treatments

When a sucrose concentration in LM media of 0.1 M or both 0.1 M and 0.2 M was used in the thawing procedure, the survival rate of ETs following cryopreservation was 75%; when samples were placed directly on the proliferation media (0.03 M sucrose), the survival rate was 50%. There were differences between the lines in the response (Table 2). For example, three of the lines showed no regrowth at all.

Of 36 samples placed on 0.1 M sucrose media, 18 grew vigorously, and 9 samples were alive but growing slowly. When the samples went through two different sucrose concentrations, the number of vigorously and slowly growing samples was vice versa, 9 and 18, and when the samples were placed directly on the proliferation media, 11 grew vigorously, and 4 slowly.

When the samples were placed directly on the proliferation media, only five lines’ (655, 809, 1046, 1037, and 1082) regrowth was sufficiently vigorous to have material for maturations (Table 3). Only these lines were therefore taken for maturation analysis. The embryo production capacity of the thawed SE lines showed no significant differences between treatments, being 133.01 ± 27.35 E/g FW when media with sucrose concentrations of 0.2 M and 0.1 M were used, and 130.47 ± 31.35 E/g FW when media with a sucrose concentration of only 0.1 M were used. When the samples were placed directly on the proliferation media, the embryo production capacity was 156.67 ± 35.07 E/g FW. Before the cryopreservation experiments, the mean embryo production capacity of the same five lines was 143.60 ± 48.50 E/g FW.

### 2.4. Experiment IV to Test Modifications of Pre-Treatments

When the ET was pre-treated using sugar concentration media of both 0.1 M and 0.2 M, 85% of the samples showed regrowth, and when only sucrose media of 0.2 M was used, the survival rate was 82%. Changing PEG6000 to PEG4000 in the cryoprotectant mixture reduced the survival rate to 67%. As in previous experiments, there were differences between the lines (Table 4), but this time, only one line did not show regrowth at all.

Seven vigorously growing SE lines (809, 1037, 1041, 1082, 1083, 1119, and 1129) were used in the maturations, of which line 1037 did not produce any embryos and therefore it was left out of statistical testing (Table 5). When 0.1 M and 0.2 M sucrose concentrations were used in the LM pre-treatment media, the embryo productivity was 62.34 ± 16.19 E/g FW, not differing from that of 61.76 ± 16.85 E/g FW when only a sucrose concentration of 0.2 M was used. When PEG4000 was used in the pre-treatment media, the embryo productivity was lower, i.e., 44.76 ± 10.51, but the difference was not significant. Before the cryopreservation experiments, the embryo capacity of the same seven lines was 112.14 ± 59.48 E/g FW.

## 3. Discussion

Storing a large number of Norway spruce genotypes in the cryo-bank enables the selection of productive SE lines for mass propagation without a loss of genetic diversity [6]. For future mass production, several ET samples from recently initiated SE lines should be cryopreserved for a short period to save the embryogenic productivity of ET [8,28]. This requires a well-refined cryo-protocol, especially when working with numerous SE lines, as exemplified in the Finnish SE Program, with the annual number of cryo-stored samples ranging from 5000 to 10,000. The selection of productive SE lines, including thawing of the ET from a large number of genotypes, also requires labor and material resources. Fine-tuning the cryo-protocol may therefore prove to be a valuable asset in achieving cost-effective mass propagation while maintaining genetic diversity. In this study, pre- and post-treatments of the cryopreservation protocol were simplified without any loss in SE line regeneration or somatic embryo production capacity. However, efforts to replace cryopreservation with storing at −80 °C to save valuable LN led to a drastic decrease in survival.

Reactive-oxygen-species (ROS)-induced oxidative stress is recognized to be a major problem in successful cryopreservation [29]. ROS is known to activate different protein kinase signaling to regulate normal physiological functions [30]. ABA biosynthesis is also upregulated in response to low temperature and drought, and increased ABA levels can partly activate the same protein kinase pathways (rev. [31]). ABA is known to be an antioxidant protecting against oxidative stress and it has been demonstrated to be one exogenous application to improve cryopreservation success [26,29]. In a recent study using garlic (*Allium sativum* L.) and *Arabidopsisi thaliana*, Xing et al. [32] discovered that ABA induced the expression of AsKIN, which increased the expression level of genes related to cold and osmotic stress, enhanced the tolerance to oxidative stress during cryopreservation and promoted plant growth. However, Edesi et al., 2020 [33] reported that a preculture of the explant with ABA significantly decreased shoot regrowth in cryopreserved buds of *Rubus humulifolius*, which is more in line with our results. When used in maturation media, it has a positive effect in promoting the maturation of embryogenic tissues, but it can also inhibit the germination and height growth of emblings for several growing seasons after exposure [34].

Reaction pathways in plants are complex and besides activators, such as ROS and ABA, and calcium accumulation in the membrane, several mediators are involved in the next step of a plant’s response to abiotic stress (fig 2, [30]). ABA-independent signaling pathways have originally been detected in *Arabidopsis*, but homologues to dehydration-responsive element-binding proteins DREB1a-c (also called CBF1-3), which respond to temperatures under +4 °C, and DREB2, which induce the expression of osmotic stress responsive genes, have been identified in conifers, such as white spruce (*Picea glauca* (Moench) Voss) [35] and Sitka spruce (*Picea sitchensis* (Bong.) Carr.) [36]. However, clear evidence of the CBF pathway is lacking in conifers. When compared to previous cryopreservation experiments using Norway spruce ET [21], our divergent results may indicate that there are also reaction pathways other than ABA-dependent pathways.

Storing in an ultra-low temperature freezer at −80 °C using a routine pre-treatment method in semi-solid media gave surprisingly low regeneration rates compared to storing in LN. Storage at −80 °C of ET has only been reported for *P. radiata* [27], although this storage temperature has been used for other types of germplasm, such as the pollen of different angiosperms [37,38], or macroalgal tissues [39]. The complexity of the ET and its high-water content may hinder the application of preservation at this temperature for Norway spruce ET. In radiata pine, the regeneration rates with this type of storage were acceptable for up to one year. However, after that period, they started to decline (Montalbán, personal communication). This is in line with the assumption that temperatures that are above those reached with LN allow the continuation of chemical processes. However, this hypothesis is not supported by the other finding of this study, i.e., the equal decrease in regeneration rates, five percentage points, after seven months of storage both at −80 °C and in LN. One reason for the decline could be the fluctuation in sample temperature when the previous sample lot was thawed from the same storing boxes, potentially causing unwanted problems with ice crystallization. Storing at −80 °C could primarily be used for the screening of the lines and may be applicable in long-term storage after refining.

Liquid media have been used successfully in the cryopreservation of Norway spruce ET [14,16,25], but with Finnish material, suspension cultures have also been less successful than semi-solid media in previous experiments [8]. The proliferation of the ET in the suspension culture has been as successful as in semi-solid media, although with remarkable genotypic variation and a lower number of embryos produced unless the ET from suspension culture was rinsed with liquid media [40]. The rotation of suspension culture may expose cells, especially suspensors to damage or additional stress. In the case of a study of radiata pine [27], the effect of liquid preculture was tested before cooling, and all the cell lines tested regrew after suspension culture. The success with Mr. Frosty freezing containers was variable in the writers’ previous research [8], when the recovery percentage varied from 7 to 100. In this study, the effect of using Mr. Frosty is difficult to evaluate due to the total failure in sample regeneration in both treatments.

Previously, pre-treatment with two elevated sucrose concentrations in the media has been used successfully in the cryopreservation of ETs of Norway spruce [8] and, e.g., *Pinus sylvestris* [41,42] and *Abies cephalonica* [43,44]. Recently, a prolonged preculture on four semi-solid media with increasing sucrose concentrations, combined with air-drying, has also been shown to result in successful cryopreservation even without using any cryoprotectants, both in *P. omorika* [20] and in *P. abies* [21] However, the writers’ experiment using a longer pre-treatment process was unsuccessful. The regeneration percentage fell to 58 from 96 in the controls when 0.4M sucrose medium was added for 24 h, and the sucrose content of mLM liquid in cryovials was increased to 0.8 M (unpublished data). A short pre-treatment using liquid media supplemented with 0.4M sorbitol has been used successfully, at least for interior spruce (*Picea glauca–engelmannii* complex) [13], western white pine (*Pinus monticola* Dougl.) [45], and maritime pine (*Pinus pinaster*) [46]. Salaj et al. 2011 [47] tested one semi-solid pre-treatment medium with 0.5 M sucrose, maltose, or sorbitol concentration with *Pinus nigra* Arn. ET and found no differences between sucrose and maltose. However, sorbitol gave lower recovery frequencies. Cryopreservation without any pre-treatment has not been tested, and further studies are needed to determine if dehydration using the elevated sucrose concentration only in cryovials is enough to avoid the crystallization of intracellular water in Norway spruce ET.

The lower regeneration rate after using PEG4000 instead of PEG6000 in cryoprotectant solution in cryovials was unexpected and unwanted. Little is known about the actual role of PEG in cryopreservation, but it has been demonstrated that in sugarcane suspensions, PEG6000 may counteract the toxicity of other cryoprotectants [48], and Finkle et al. [49] suggested that it helped maintain the membrane structures. It may be that the lower molecular weight of PEG4000 compared to PEG6000 affects the outcome of the protection process.

The most common thawing procedure is to place the ET on filter paper and then place it on proliferation media and change it to fresh media once or twice in various cycles [13,14,15,16]. Media with the same sucrose content as in the pre-treatment but in reverse order have been used with Scots pine (*Pinus sylvestris*) [36], Serbian spruce (*Picea omorica*) [20], and Norway spruce [8,21]. In this study, the best regeneration of ET was achieved when only one medium with elevated sucrose was used after thawing, and although the differences were not significant, and the best embryo productivity was achieved when only proliferation media were used. This may refer to better osmotic regulation in the quick rehydration of the ET and perhaps to the avoidance of an unwanted continuation of dehydration.

The growth rates of the ET after cryopreservation varied between genotypes and samples within the genotype. Unfortunately, some of the ET continued growing slowly, which creates a problem with statistical testing and evaluation of the usability of the genotype in a mass propagation program.

In conclusion, the present study showed that both pre-and post-treatments on the semi-solid media in the cryopreservation protocol of Norway spruce ETs could be simplified by reducing the number of media, without any loss of genotype or the embryo production capacity of the ETs. On the contrary, the storage of the ETs in a freezer at −80 °C instead of in LN is not possible, and the addition of ABA in the pre-treatment media does not provide benefits but increases costs. The simplified pre-and post-treatment protocol will reduce workload and costs remarkably in the mass cryopreservation of future forest regeneration materials and in thawing the samples for mass propagations, respectively.

## 4. Materials and Methods

### 4.1. Plant Material

Embryogenic lines of Norway spruce were initiated from immature seed embryos originating from controlled crossings of a Finnish tree breeding program or from open pollinated special forms for ornamental use (Table 6). Crossings were made in 2012 and 2014, using seed orchard grafts in southern Finland (60°55′ N, 26°13′ E, 80 m, 60°41′ N, 24°02′ E, 130 m). Grafted trees and pollen donors originated from different locations in southern Finland. In 2020, cones were collected from two open-pollinated special forms, i.e., from a tree with a globular crown (*Picea abies* f. *globosa*) and a graft of a broom-shaped Norway spruce (*P. abies* f *condensata*).

### 4.2. Initiation and Proliferation of the SE Lines

Immature cones were collected in the summer, when the heat sum was approximately 800 dd. The cones were cleaned with 70% ethanol, the seeds were dissected in sterile water, and dishwashing soap was added to clean the seeds. After one rinse in sterile water, the seeds were surface-sterilized in 70% ethanol for 5 min and rinsed three times in sterilized water. Zygotic embryos were dissected from megagametophytes and placed on modified Litvay’s medium (mLM) containing half-strength macro-elements [50] and 10 µM 2,4-dicholophenoxyacetic acid (2.4-D) (Alfa Aesar, Haverhill, MA, USA) and 5 µM 6-benzyladenine (BA) (Sigma Aldrich, Saint Luis, MO, USA) as plant growth regulators. The sucrose (VWR, Radnor, PA, USA) concentration of media was 0.03 M. The pH of the medium was adjusted to 5.8 prior to adding gelling agent (Phytagel 4 g/L (Sigma Aldrich, Saint Luis, MO, USA)) and sterilization in the autoclave. After autoclaving at 120 degrees for 20 min, the medium was cooled to 60 °C, and 500 mg/L of L-glutamine (Merck, Darmstadt, Germany) was added using filter sterilizing. Petri dishes (9 cm in diameter) were filled with 20 mL of medium.

Ten zygotic embryos were placed on the same petri dish, and they were kept in the dark and at 24 °C. The zygotic embryos stayed for two to eight weeks on the same media until the ET started to grow and were picked up onto a fresh medium, each genotype on a separate dish. Established ETs were subcultured every two weeks. The proliferation media was the same as that used for culture initiation. For the experiments, the ET was collected five to seven days following the last subculture. Embryogenic lines were chosen based on their good growth in proliferation.

### 4.3. Experimental Design

#### 4.3.1. Experiment I to Test ABA in the Pre-Treatment Media

Pre-treatment according to Varis et al. [8] was used as a control, i.e., 24 h growth of clumps of ET on semi-solid mLM media with 0.1 M and 0.2 M sucrose content, slow-cooling in a programmable cooling device, and storing in LN. In the experiment, 10 µM ABA (Sigma Aldrich, Saint Luis, MO, USA) was added to both pre-treatment media. The nine different genotypes were from different crossings, in which nine mother and father genotypes were used (Table 6). The experiment started in October 2013; the ETs had therefore been under in vitro culture for approx. 13 months.

After pre-treatment, about 200 mg of ET was placed in sterile cryovials (Sarstedt, Nümbrecht, Germany)(six to eight clumps in each 2 mL cryovial) containing 400 µL liquid mLM medium with a sucrose concentration of 0.4 M but without plant growth regulators, gelling agent, or glutamine. Cryovials were placed on CoolRacks^®^ (Corning, Glendale, CA, USA) which were pre-cooled at −20 °C. To each cryovial 400 µL pre-chilled cryoprotectant, PGD solution (composed of polyethylene glycol 6000 (VWR, Radnor, PA, USA), glucose (Merck, Darmstadt, Germany), and Me_2_SO 10% *w/v* each) was added in 200 µL aliquots during the half-hour period. The cryovials were then incubated for half an hour in cooling racks. The samples were frozen with a slow-cooling method using a programmable cooling device (Planer, Kryo 10 series III, Planer Products, Middlesex, UK). The samples were frozen in the Planer by −0.17 °C/min to −38 °C. Three cryovials containing ET from the same genotype as the control and experimental media were stored in LN.

After two weeks storage in LN, the samples were thawed in a water bath at +37 °C for 2 min. The cryovials were wiped with 70% ethanol, and the content of the tube was poured into the sterilized paper filter (Whatman #2, Whatman International Limited, Kent, UK) placed in the Büchner funnel. The cryostorage liquid was drained off by suction, and the tissue was washed with the same liquid mLM medium that was used in the first pre-treatment process (sugar concentration 0.4 M). The samples were placed on a medium with a sucrose content of 0.2 M and transferred every 24 h onto media with decreasing sucrose concentrations (0.1 M and 0.03 M). All filters with tissue were transferred onto new media every two weeks. A visual observation of the regrowth of embryogenic tissue was made, and the recovery percentages were calculated.

#### 4.3.2. Experiment II to Test Storage at −80 °C

Two pre-treatment, two slow-cooling methods, and two storing temperatures were tested in four combinations: (1) a control as in the previous experiments; (2) the same pre-treatment and slow-cooling methods but storing at −80 °C; (3) pre-treatment in suspension, slow-cooling in a Mr. Frosty container, and storing at −80 °C [25]; and (4) pre-treatment in suspension, slow-cooling in the Planer before storing at −80 °C.

The experiment started in October 2020, using a total of thirteen embryogenic lines (Table 6), from which three originated in two crossings made in 2014, cryopreserved and thawed according to Varis et al. [8] in December 2019, i.e., at the time of the experiment, the ETs had been under in vitro culture for approx. 10 months. Ten of the lines were from open-pollinated special forms initiated in August 2020, so they had been in the culture for approx. 3 months. Six cryovials containing ET from the same genotype was stored from every treatment combination either in LN or in a low-temperature freezer at −80 °C.

In the first pre-treatment method, clumps of fresh ET were cultured on solid mLM media that contained increasing concentrations of sucrose (0.1 M for 24 h followed by 0.2 M for another 24 h). A slightly modified method of Montalbán and Moncaleán [26] was used to pre-culture ET from the same lines in liquid mLM medium with a sucrose concentration of 0.53 M. The pH was adjusted to 5.7 before autoclaving. Fresh ET (1.5 g) was suspended in 5.4 mL of liquid medium in a 100 mL Erlenmeyer flask. The suspension was incubated for one hour at 23 °C on an orbital shaker at 120 rpm in the dark. After incubation, the same volume of liquid medium containing 0.53M sucrose plus 15% Me_2_SO was gradually added (3 times 1.8 mL in 15 min intervals).

The suspension was pipetted into cryovials (six times 1.8 mL); and 6 cryovials, each containing 250 mg fresh mass of ET, were arranged in a Mr. Frosty container (NalgeneTM, Nalge Nunc International Corporation, Rochester, NY, USA) and in a freezer at −80 °C. According to the manufacturer, freezing in Mr. Frosty takes place at a rate of −1 °C/min. After 90 min, the cryovials with cell suspension were rapidly removed from the Mr. Frosty container and plunged into liquid nitrogen for 2 min, and the cryovials were then stored in the freezer (−80 °C). The same amount of cryovials from each genotype was pre-treated in the same way but placed on the slow-cooling Planer device before storing in the freezer.

After 3 months, three samples from each line were taken from LN and at −80 °C and immediately thawed in a water bath at +37 °C for 2 min. An equal number of samples was thawed ten months after storing.

#### 4.3.3. Experiment III to Test Reduction of Post-Thawing Treatments

To test the simplification of the thawing process, vigorously growing ET from three-month cryopreserved control samples from the previous experiment was cryopreserved again using the same method, i.e., pre-treatment on semi-solid media with a sucrose content of 0.1 M and 0.2 M, slow-freezing in the Planer, and storage in LN. The samples were thawed after a four-week storage in LN according to Varis et al. [8]. The samples were thawed in a water bath at +37 °C for 2 min. The cryovials were wiped with 70% ethanol, and the content of the tube was poured onto sterilized paper filter (Whatman #2, Whatman International Limited, Kent, UK) and placed in the Büchner funnel. The cryostorage liquid was drained off by suction, and the tissue was washed with the same liquid mLM medium that was used in the pre-treatment process. Post-thawing treatment was performed using three different procedures, with media with different sucrose concentrations: (1) the same as in the previous experiments, i.e., the samples placed first on medium with a sucrose content of 0.2 M and transferred every 24 h onto media with a decreasing sucrose concentration (0.1 M and 0.03 M); (2) samples placed on 0.1 M sucrose concentration for 24 h and then transferred onto proliferation media (0.03 M sucrose); and (3) placing filters with tissue directly onto proliferation media and transferring onto new proliferation media after 24 h.

The thawing experiment was performed in March 2021, using ten embryogenic lines originating in five crossings made in 2014 and from open-pollinated special forms initiated in 2020 (Table 6). The lines initiated in 2014 were cryopreserved and thawed several times. The latest thawing was in November 2020, meaning that at the time of the experiment, the ETs had been under culture for approximately four months. The ET from SE lines initiated in 2020 were the control samples from the experiment’s test storing at −80 °C. They were thus cryopreserved and thawed once. Those lines were thawed in February 2021, so at the time of the experiment, the ETs had been under culture for one month.

From every line, three cryovials per treatment were cryopreserved. All filters with thawed tissue were transferred onto new media every two weeks. A visual observation of the regrowth of embryogenic tissue was made, and the recovery percentages were calculated. Vigorously growing ET from seven lines was maturated.

#### 4.3.4. Experiment IV to Test Modifications of Pre-Treatments

In this experiment, three combinations of pre-treatments were used. First, to test the effect of shortened pre-treatment on the success of cryopreservation, the ET was pre-treated only on the solid media containing 0.2 M sucrose (24 h). Second, due to availability problems, PEG 6000 in PDG was replaced with 6.6% *w/v* PEG4000 (Merck, Darmstadt, Germany). To keep the number of PEG molecules per liquid unit at the same level as PEG6000, the mass of PEG4000 was reduced (n = m/M, PEG6000 10 g/6000 g/mol = 0.001666 mol, 10 g/4000 g/mol = 0.0025, and 4000 g/mol × 0.001666 mol = 6.66 g). Third, pre-treatment used in previous experiments, i.e., 24 h in 0.1 M and again 24 h in 0.2 M, was used as a control method. PDG cryoprotectant mixtures were added, and slow-cooling in the Planer was used. The samples were thawed after two weeks of storing. In thawing, only media with a concentration of 0.1 M sucrose before proliferation media was used.

The pre-treatment experiment was performed in October 2021, using 11 embryogenic lines originating in one crossing made in 2014 and from open-pollinated special forms initiated in 2020. The lines were the same as in the experiment’s test storing at −80 °C. The lines initiated in 2014 were thus cryopreserved and thawed twice; the lines initiated in 2020 were cryopreserved and thawed once. All lines were thawed in August 2021, i.e., at the time of the experiment, the ETs had been under culture for approximately two months. From every line, three cryovials per combination were frozen. All filters with tissue were transferred onto new media every two weeks. A visual observation of the regrowth of embryogenic tissue was made, and the recovery percentages were calculated. The recovery percentage was counted for all the samples that had growth and vigorously growing samples. Vigorously growing ET from seven lines was maturated.

### 4.4. Maturation

To test the effects of cryopreservation methods on embryo maturation capacity, the thawed and growing lines from cryopreservation Experiments II and III were maturated using a filter method modified from Lelu-Walter and co-workers [41]. All the lines were also maturated before Experiment I started. Five to seven days after the last subculture, about 180 (±20) mg of embryogenic tissue was mixed in 3 mL liquid mLM without plant growth regulators (PGR), and the suspension was poured onto paper filter (Whatman #2) placed in the Buchner funnel. The liquid was drained off by suction, and the filter was placed on mLM medium with 60 µM abscisic acid (ABA) and 0.2 M sucrose, gelled with six g/l of Phytagel. An appropriate aliquot of filter-sterilized stock solution of ABA was added to the medium after autoclaving. After eight weeks, the number of cotyledonary embryos per gram of fresh weight (E/g FW) was counted for three dishes per line.

### 4.5. Statistical Analyses

The number of embryos produced was compared using the independent samples t-test for two grouping variables and one-way ANOVA for three independent groups. Differences were considered significant at the 5% level. The mean values are presented with the standard error. All the statistical analyses were performed using IBM SPSS Statistics 22.0.

## 5. Conclusions

Both pre-and post-treatments on semi-solid media can be simplified by reducing the number of media, without any loss of genotype or embryo production capacity of ETs. On the contrary, the storage of ETs in a freezer at −80 °C instead of LN is not possible, and the addition of ABA in the pre-treatment media does not provide benefits but increases costs. The lower regeneration rate after using PEG4000 instead of PEG6000 in cryoprotectant solution in cryovials was unexpected and unwanted. The simplified pre-and post-treatment protocol will remarkably reduce workload and costs in the mass cryopreservation of future forest regeneration materials and in the thawing of the samples for mass propagations, respectively.

## Figures and Tables

**Table 1:** Number of samples regenerated after cryopreservation when pre-treated with or without ABA, and embryo production capacity per one gram of fresh weight of ET. Three cryovials containing ET from the same genotype as the control and experimental media were stored in LN.

SE Line	Regenerated Samples		Embryos/g FW	
	ABA	Control	ABA	Control
3006	0	2		
3128	0	2		
3301	0	0		
4310	2	3	151.76 ± 8.88	165.76 ± 15.53
5852	2	3	1.90 ± 1.90	0
4611	3	3	18.29 ± 10.88	0
4934	2	2		
6375	3	3	12.82 ± 4.87	27.54 ± 1.72
9130	3	3	98.51 ± 8.33	115.46 ± 36.48
Total	15	21	56.66 ± 15.94	61.72 ± 19.14

**Table 2:** Number of samples regenerated after cryopreservation and different thawing treatments. The three treatments were 24 h both in 0.2 M and 0.1 M sugar concentration LM media before the proliferation media (0.03 M sucrose), 24 h in a 0.1 M sucrose concentration before proliferation media and placing samples directly on LM proliferation media (0.03 M sucrose concentration). Three cryovials containing embryogenic tissue from the same genotype from every experimental media were stored in LN.

SE Line	0.2 M and 0.1 M Sucrose			0.1 M Sucrose			0.03 M Sucrose		
	Growing	Slow Growing	Dead	Growing	Slow Growing	Dead	Growing	Slow Growing	Dead
655	3			3			3		
809	3			3			2	1	
1037		3		3			3		
1046		3		2	1		2	1	
1082		3			3		1	2	
1119 *		3		2	1				
1129 *	3			3					
1130		3		2	1				3
2851			3			3			3
4262			3			3			3
5111			3			3			3
5129		3			3				3
Total	9	18	9	18	9	9	11	4	15

* 0.03 M sucrose concentration media was not included.

**Table 3:** Embryo production capacity per one gram of fresh weight of embryogenic tissue. Three cryovials containing ET from the same genotype from every experimental media were stored in LN and three maturations were made from regrowth from every cryovial.

SE Line	Embryos/g FW		
	0.2 M and 0.1 M Sucrose	0.1 M Sucrose	0.03 M Sucrose
655	155.84 ± 4,00	122.39 ± 17.18	307.50 ± 13.07
809	249.90 ± 78.09	345.15 ± 19.23	258.41 ± 97.28
1037	0.72 ± 0.72	1.45 ± 1.45	0.73 ± 0.73
1046	87.43 ± 13.27	67.03 ± 9.59	88.34 ± 31.25
1082	171.14 ± 47.84	116.35 ± 11.55	128.38 ± 31.35
Total	133.01 ±27.35	130.47 ± 31.35	156.67 ± 35.07

**Table 4:** Number of samples regenerated after different pre-treatments and cryopreservation in Experiment III. The pre-treatments were 24 h in both 0.2 M and 0.1 M sugar concentration mLM media or 24 h in 0.2 M sucrose concentration mLM medium. The two-step sucrose treatment with PEG4000 instead of PEG6000 in a cryoprotectant solution (composed of PEG, glucose, and Me_2_SO) was also tested. In thawing, the samples were first placed on 0.1 M sucrose media for 24 h before proliferation media (0.03 M sucrose concentration). Three cryovials containing ET from the same genotype from every experimental medium were stored in LN.

SE Line	0.1 M and 0.2 M Sucrose and PEG6000			0.2 M Sucrose and PEG6000			0.1 M and 0.2 M Sucrose and PEG4000		
	Growing	Slow Growing	Dead	Growing	Slow Growing	Dead	Growing	Slow Growing	Dead
809	3			3			3		
894	2		1	1		2		1	2
1037	3			3			3		
1041	3			2	1		2	1	
1046	2		1	3					3
1050	2	1		1	1	1			3
1082	3			3			3		
1083	3			3			3		
1119	3			2	1		3		
1129	3			3			3		
1130			3			3			3
Total	27	1	5	24	3	6	20	2	11

**Table 5:** Embryo production capacity per one gram of fresh weight of embryogenic tissue. Three cryovials containing embryogenic tissue from the same genotype from every experimental media were stored in liquid nitrogen and three maturations were made from regrowth from every treatment.

SE Line	Embryos/g FW		
	0.1 M and 0.2 M Sucrose and PEG6000	0.2 M Sucrose and PEG6000	0.1 M and 0.2 M Sucrose and PEG4000
809	134.39 ± 8.70	100.23 ± 28.80	83.16 ± 54.49
1041	52.73 ± 8.03	41.12 ± 27.04	50.82 ± 9.45
1082	64.73 ± 7.24	165.99 ± 45.13	36.83 ± 7.47
1083	110.96 ± 78.06	61.01 ± 28.36	67.59 ± 21.01
1119	11.01 ± 2.24	2.19 ± 2.19	15.06 ± 8.71
1129	0.21 ± 0.21	0	15.11 ± 8.68
Total	62.34 ± 16.19	61.76 ± 16.85	44.76 ± 10.51

**Table 6:** Norway spruce SE lines used in the experiment using ABA in the pre-treatment media (I) storing embryogenic tissue at −80 °C (II), one post-thawing medium compared to two (III), and one pre-treatment compared to two (IV). The initiation years and origin (full-sib family or mother tree), embryo production capacity (embryos/g Fresh Weight), as well as the cryopreservation and thawing status of the line in each experiment, are shown.

Experiment	SE Line	Initiation Year	Origin	Embryos/g FW before Experiment
I	3006	2012	E2515 × K805	206
**I**	3128	2012	E2853 × E231	78
**I**	3301	2012	E329 × E2089	60
**I**	4310	2012	E2853 × E330	327
**I**	5852	2012	K264 × E330	0
**I**	4611	2012	K264 × E231	40
**I**	4934	2012	E318 × E231	4
**I**	6375	2012	E318 × K805	12
**I**	9130	2012	E329 × K805	61
II	290	2014	E162 × E81	4
II	1048	2020	Open-pollinated *Pa f. condensata*	0
II, III *, IV *	809	2020	Open-pollinated *Pa f. globosa*	418
II, III *, IV *	1037	2020	Open-pollinated *Pa f. condensata*	0
II, III *, IV *	1046	2020	Open-pollinated *Pa f. condensata*	198
II, III *, IV *	1082	2020	Open-pollinated *Pa f. condensata*	223
II, III *, IV *	1119	2020	Open-pollinated *Pa f. condensata*	0
II, III *, IV *	1129	2020	Open-pollinated *Pa f. condensata*	0
II *, IV **	1041	2014	E207 × E1373	42
II *, IV **	1050	2014	E207 × E1373	75
II, IV *	894	2020	Open-pollinated *Pa f. globosa*	15
II, IV *	1083	2020	Open-pollinated *Pa f. condensata*	102
II, IV *	1130	2020	Open-pollinated *Pa f. condensata*	11
III ****	655	2014	E18 × E436	330
III ***	1130	2014	E207 × E1373	191
III ****	2851	2014	E46 × E3222	405
III ***	4262	2014	E9 × E3231	310
III ***	5111	2014	E799 × E1366	106
III ***	5129	2014	E799 × E1366	307

* = cryopreserved and thawed once. ** = cryopreserved and thawed twice. *** = cryopreserved and thawed three times. **** = cryopreserved and thawed four times.

## Data Availability

The raw data supporting the conclusions of this article will be made available by the authors, without undue reservation.
